# Widespread disruptive selection in the wild is associated with intense resource competition

**DOI:** 10.1186/1471-2148-12-136

**Published:** 2012-08-02

**Authors:** Ryan A Martin, David W Pfennig

**Affiliations:** 1 Department of Biology, CB#3280, University of North Carolina, Chapel Hill, NC 27599, USA; 2 Present address: Department of Biology, CB#7617, North Carolina State University, Raleigh, NC 27695, USA

**Keywords:** Competition, Disruptive selection, Resource polymorphism, Selection differential, Spadefoot toad, Quadratic selection

## Abstract

**Background:**

Disruptive selection has been documented in a growing number of natural populations. Yet, its prevalence within individual systems remains unclear. Furthermore, few studies have sought to identify the ecological factors that promote disruptive selection in the wild. To address these issues, we surveyed 15 populations of Mexican spadefoot toad tadpoles, *Spea multiplicata,* and measured the prevalence of disruptive selection acting on resource-use phenotypes. We also evaluated the relationship between the strength of disruptive selection and the intensity of intraspecific competition—an ecological agent hypothesized to be an important driver of disruptive selection.

**Results:**

Disruptive selection was the predominant mode of quadratic selection across all populations. However, a directional component of selection favoring an extreme ecomorph—a distinctive carnivore morph—was also common. Disruptive selection was strongest in populations experiencing the most intense intraspecific competition, whereas stabilizing selection was only found in populations experiencing relatively weak intraspecific competition.

**Conclusions:**

Disruptive selection can be common in natural populations. Intraspecific competition for resources may be a key driver of such selection.

## Background

Disruptive selection occurs in a population when two or more modal phenotypes have higher fitness than the intermediate phenotypes between them [1]. Disruptive selection has long been viewed as important in maintaining and increasing variation within natural populations [1-3]; favoring the evolution of alternative phenotypes [4,5] and sexual dimorphism [6-8]; and even initiating speciation [6,8-12]. Nevertheless, compared to the other main modes of selection—directional selection and stabilizing selection—disruptive selection has traditionally received much less attention.

Despite this relative lack of attention given to disruptive selection, recent meta-analyses suggest that disruptive selection may be at least as common as stabilizing selection [13,14]. Indeed, an increasing number of studies have documented disruptive selection in natural populations [15-24]. Yet, although these data imply that disruptive selection may be widespread, we still do not know how prevalent it can be within individual systems where it has been found to occur [22].

Additionally, the actual causes of disruptive selection have been relatively understudied. Longstanding theory suggests that disruptive selection arises from negative frequency-dependent interactions, such as those stemming from intraspecific competition, predation, mutualism, and parasitism [10,25-28]. To illustrate how intraspecific competition generates disruptive selection [3,8,10,29,30], consider first that—owing to functional trade-offs [17-20,31-35]—individuals with certain phenotypes are generally better adapted than are individuals with other phenotypes at utilizing specific resource types [36]. Yet, as any one of these modal phenotypes becomes common, it will tend to suffer from resource depletion, as these individuals compete more against themselves than against other resource-use phenotypes in the same population [18,37]. Consequently, the less common modal phenotype(s) will be favored, because they will experience reduced competition for their resources [10,35,38]. In this way, intraspecific competition acts as an agent of frequency dependent disruptive selection, which favors two or more modal resource-use phenotypes in the same population [35,38,39].

Empirical studies largely support this theory. Indeed, intraspecific competition has been shown to cause disruptive selection in natural populations of three-spine sticklebacks [22,35], spadefoot toad tadpoles [18,21], and Eurasian perch [23], and it has been implicated as causing disruptive selection in various other systems [15-17,19,20,40]. Moreover, several studies have shown that competition generates negative frequency-dependence among different resource-use phenotypes [31,41-44], which (as noted above) is a hallmark of competitively mediated disruptive selection [38].

Nevertheless, relatively few comprehensive surveys of natural populations have sought to identify the ecological conditions that are associated with disruptive selection [22,23]. For example, the relationship between spatial variation in the strength of intraspecific resource competition and the occurrence and intensity of disruptive selection has been little examined in un-manipulated wild populations. This relationship has likely been difficult to evaluate because doing so requires evaluating selection in replicated populations, some of which experience weak resource competition and some of which experience strong resource competition.

We sought to fill these gaps in our knowledge concerning the prevalence and correlates of disruptive selection in natural populations. We specifically tested two predictions: first, that disruptive selection may be common within certain systems, and second, that disruptive selection would be more intense under conditions of greater resource competition. We tested these two predictions in natural populations of Mexican spadefoot toads (*Spea multiplicata*).

Spadefoot toad tadpoles are well-suited for such studies, because they express a remarkable range of trophic phenotypes in the wild [45,46]. The extremes of this variation are represented by two ecomorphs that comprise a resource polymorphism: an “omnivore” morph—a round-bodied tadpole with a long intestine, small jaw muscles, numerous rows of keratinized denticles, and smooth keratinized mouthparts that feeds primarily on the pond bottom, and a “carnivore” morph—a narrow-bodied tadpole with a short intestine, greatly enlarged jaw muscles, few rows of keratinized denticles, and notched, serrated keratinized mouthparts that feeds mostly in the water column [18,47,48]. Omnivores are generalists, which feed mostly on microscopic detritus, algae, and small crustaceans, whereas carnivores are specialists, which feed mostly on anostracan fairy shrimp [37,49].

Although carnivore development is induced by consumption of fairy shrimp [50], heritable variation for morph development exists within natural populations [48,51]. The degree to which a population expresses this resource polymorphism varies across ponds, and is, in part, associated with variation in conspecific density and ecological opportunity (i.e., the presence of underutilized, accessible resources). Specifically, bimodality in trophic phenotypes is greatest in ponds where conspecific density and ecological opportunity are highest [46].

Previous research suggests that intermediate phenotypes are disfavored by disruptive selection in this system [18,21,46]. In particular, compared to tadpoles with intermediate phenotypes, omnivores and carnivores are larger, more developmentally advanced, and more likely to survive to metamorphosis [18,21]. Furthermore, previous experiments have shown that this disruptive selection reflects negative frequency-dependent interactions driven by ecological specialization and resource competition [43,46].

**Figure 1:**
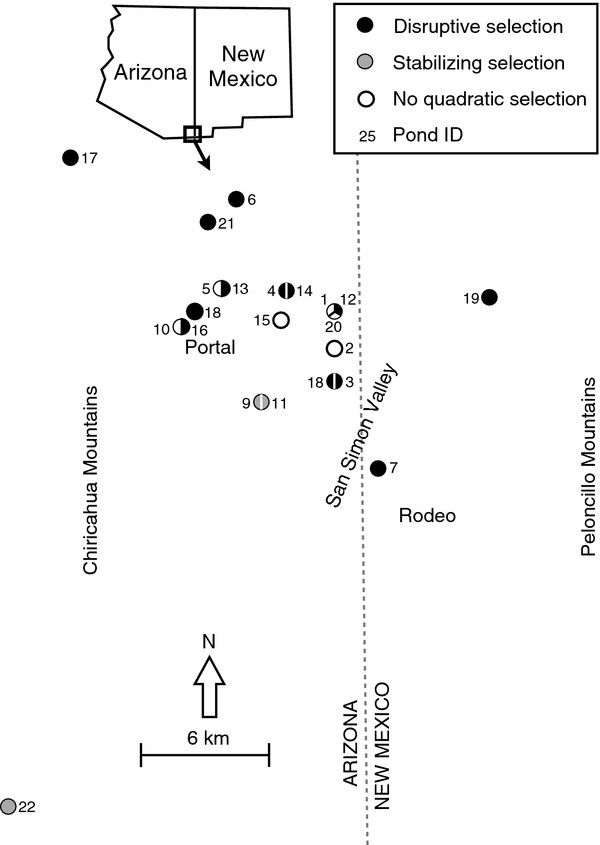
**Localities.** Map of study area illustrating locations of ponds sampled and the form of quadratic selection in each pond. Symbols for ponds sampled in multiple years are divided to show the form of quadratic selection in each year. The numbers beside each symbol section correspond to each collection’s pond ID referenced as “Map ID” in Tables.

However, it is unclear how prevalent disruptive selection is among populations of *S. multiplicata* or how ecological variation impacts the mode and strength of selection in this system. We therefore addressed these issues by measuring the mode and magnitude of phenotypic selection, as well as the strength of intraspecific competition within natural populations. We focused on populations of Mexican spadefoot toad tadpoles (*S. multiplicata*) in the San Simon Valley of southeastern Arizona and southwestern New Mexico, USA (Figure 1). These populations are ideal for addressing the above issues because the *S. multiplicata* in these populations express a resource polymorphism that is thought to have arisen from disruptive selection [18,21,46]. Moreover, previous research suggests that disruptive selection may be widespread in these populations [18,21]. Finally, the *S. multiplicata* in these populations experience a wide array of ecological conditions over a small geographic area [21,52,53]*.*

We made *a priori* predictions based on theory as well as the prior work on this system (see above). Specifically, we predicted that disruptive selection on tadpole trophic morphology would be widespread in our surveyed populations. We also predicted that this disruptive selection would be strongest in ponds where intraspecific competition is the most intense (as measured by conspecific density and per capita resource abundance).

## Results

### Evaluating the prevalence of disruptive selection

**Figure 2:**
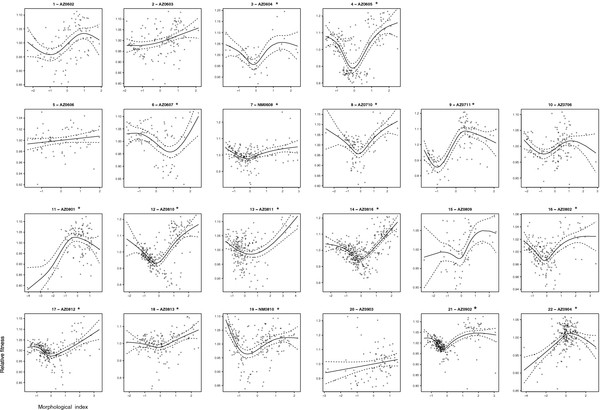
**Fitness functions.** Cubic-splines of relative fitness (measured by tadpole body size) on a composite shape variable of trophic morphology. The cubic spline (solid line) is bracketed by 95% confidence intervals (dashed lines). Individual panel legends correspond to the populations’ map ID followed by the pond ID. An asterisk indicates a significant fit of a quadratic regression, as well as a fitness minimum or maximum.

Disruptive selection was the predominant mode of quadratic selection on tadpole trophic morphology (Figure 1). Indeed, disruptive selection, identified by a significantly positive γ (Table 1) and a fitness minimum (Figure 2), occurred at least once in 73% of the unique ponds sampled (11 of 15 unique ponds) and in 59% of our total collections (Table 1; 13 of 22 collections). In contrast, stabilizing selection, identified by a significantly negative γ (Table 1) and a fitness maximum (Figure 2), occurred in 13% of the unique ponds sampled (2 of 15 unique ponds) and in 14% of our total pond collections (Figure 1, Table 1; 3 of 22 collections). Additionally, a significant directional component of selection favoring carnivore-like phenotypes was present in 80% of the unique ponds sampled (Table 1; 12 of 15 unique ponds) and 82% of total pond collections (Table 1; 18 of 22 collections).

**Table 1:** The mode and strength of selection on trophic morphology in natural ponds

**Pond**	**Map ID**	**Year**	**β/γ**	**Selection differentials**	**SE/2SE**	***t***	***P***
AZ0602	1	2006	β	**.022**	**.006**	**3.678**	**.0003**
			γ	.013	.012	1.181	.240
AZ0603	2	2006	β	**.020**	**.005**	**3.667**	**.0003**
			γ	.019	.011	1.639	.103
AZ0604	3	2006	β	.014	.008	1.622	.111
			γ	**.039**	**.013**	**2.874**	**.006**
AZ0605	4	2006	β	**.031**	**.009**	**3.487**	**.0006**
			γ	**.126**	**.014**	**8.258**	**<.0001**
AZ0606	5	2006	β	.003	.002	1.423	.159
			γ	-.001	.004	-.170	.866
AZ0607	6	2006	β	**-.017**	**.007**	**-2.354**	**.020**
			γ	**.038**	**.012**	**2.920**	**.004**
NM0608	7	2006	β	**.009**	**.004**	**2.253**	**.025**
			γ	**.024**	**.006**	**3.596**	**.0004**
AZ0710	8	2007	β	**.026**	**.006**	**3.857**	**.0002**
			γ	**.043**	**.008**	**4.552**	**<.0001**
AZ0711	9	2007	β	**.076**	**.009**	**8.262**	**<.0001**
			γ	**-.060**	**.018**	**-3.113**	**.002**
AZ0706	10	2007	β	.006	.003	1.67	.097
			γ	.005	.005	.936	.351
AZ0801	11	2008	β	**.030**	**.006**	**4.417**	**<.0001**
			γ	**-.036**	**.008**	**-4.618**	**<.0001**
AZ0810	12	2008	β	**.042**	**.006**	**6.841**	**<.0001**
			γ	**.075**	**.012**	**6.467**	**<.0001**
AZ0811	13	2008	β	**.008**	**.003**	**2.318**	**.021**
			γ	**.024**	**.004**	**5.741**	**<.0001**
AZ0816	14	2008	β	**.029**	**.004**	**6.781**	**<.0001**
			γ	**.068**	**.006**	**11.412**	**<.0001**
AZ0809	15	2008	β	**.017**	**.006**	**2.796**	**<.007**
			γ	.009	.008	1.224	.226
AZ0802	16	2008	β	**.005**	**.001**	**2.714**	**.007**
			γ	**.011**	**.002**	**4.377**	**<.0001**
AZ0812	17	2008	β	**.009**	**.003**	**2.699**	**.007**
			γ	**.029**	**.006**	**5.557**	**<.0001**
AZ0813	18	2008	β	**.017**	**.005**	**3.095**	**.002**
			γ	**.028**	**.01**	**3.110**	**.002**
NM0810	19	2008	β	.006	.004	1.518	.131
			γ	**.033**	**.008**	**3.812**	**.0001**
AZ0903	20	2009	β	**.020**	**.012**	**1.638**	**.105**
			γ	**.001**	**.020**	**.089**	**.929**
AZ0902	21	2009	β	**.017**	**.002**	**6.017**	**<.0001**
			γ	**.016**	**.006**	**2.692**	**.007**
AZ0904	22	2009	β	**.012**	**.002**	**5.326**	**<.0001**
			γ	**-.010**	**.002**	**-4.917**	**<.0001**

The pond name, map ID corresponding to Figure 1, and year of collection are given for each population, along with the regression terms (β/γ), estimated selection differential for each term, its standard error (SE), *t*-statistic and probability of rejecting the null hypothesis that the estimated differential is zero. For quadratic regressions, positive selection differentials signify disruptive selection and the quadratic regression coefficient is doubled to calculate the quadratic selection differential (γ) and the associated standard error (SE) is also doubled. Bolding signifies statistical significance.

### Evaluating whether competition predicts the strength of disruptive selection

**Figure 3:**
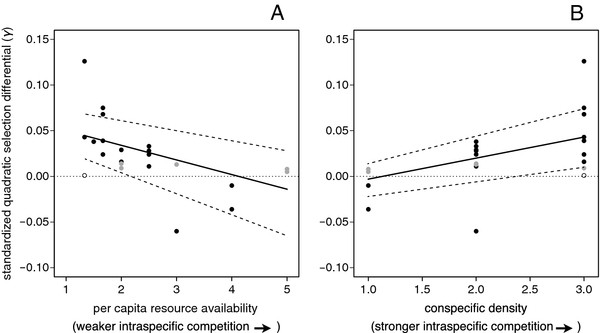
**Intensity of disruptive selection as function of the intensity of competition.** The relationship between quadratic selection and two measures of intraspecific competition. The intensity of disruptive selection on trophic morphology was greater in ponds with (**A**) lower per capita resource density and (**B**) higher conspecific density. Standardized quadratic selection differentials above the dotted line are positive and indicate disruptive selection, while those below the line are negative and indicate stabilizing selection. Significant selection differentials (black), non-significant selection differentials (grey) and significant selection differentials with no fitness minimum or maximum in the range of the data (open) are all shown. Solid and dashed lines show respectively, the median and 95% confidence intervals of the regression coefficient obtained from bootstrapping. Each point in the analysis was weighted by the populations’ sample size.

As predicted, disruptive selection was more intense in ponds with greater intraspecific competition (i.e., lower per capita resource density; Figure 3A, *F*_*1,20*_ = 9.861, *P* = .005, median regression coefficient from bootstrapping = -.016, 95% CI: -.023 − -.011, higher conspecific density; Figure 3B, *F*_*1,20*_ = 10.088, *P* = .004, median regression coefficient from bootstrapping = .023 95% CI: .016 − .030).

## Discussion

Interest in disruptive selection arises from its potential role in maintaining and accentuating variation within populations (including polymorphism) and in promoting speciation [2]. Despite this longstanding interest, the prevalence of disruptive selection in natural populations, and therefore the relative importance of disruptive selection in generating biological diversity, remains unclear. We sought to determine the prevalence of disruptive selection among natural populations of spadefoot toads. We also sought to establish whether the strength of disruptive selection was positively associated with the intensity of intraspecific competition within individual populations.

Disruptive selection favoring extreme resource-use phenotypes, as measured here by using larval body size to estimate tadpole fitness (see Methods), was widespread in the spadefoot toad populations surveyed in this study. Indeed, we documented disruptive selection on tadpole trophic morphology in 11 of 15 populations surveyed (Figure 1). These results, together with those of previous studies [18,21,46], strongly support the role of disruptive selection in the evolution of resource polymorphism in *Spea* tadpoles [46], and add to the evidence that disruptive selection can be widespread within certain systems [22].

Although disruptive selection was prevalent in our surveyed populations, we also found considerable variation in both the mode and magnitude of quadratic selection (Figures 1, 2; Table 1). This variation correlated predictably with the intensity of intraspecific competition within individual populations. Specifically, disruptive selection was strongest in ponds with the highest conspecific density and lowest per capita resource density (Figure 3A, B). In contrast, stabilizing selection favoring intermediate resource-use phenotypes was found in ponds where intraspecific competition was weak (Figure 3A, B). These results therefore confirm theory suggesting that intraspecific competition can drive disruptive selection (see Introduction). Furthermore, our results suggest that disruptive selection is likely to occur in habitats relatively free of predators, heterospecific competitors, or other ecological factors that depress population size and that therefore potentially weaken intraspecific competition.

Interestingly, the two extreme ecomorphs do not appear to be equally favored by disruptive selection. Previous studies found that carnivores achieve greater survival and are generally (although not always) larger at metamorphosis than either intermediates or omnivores [18,21]. We similarly found that a directional component to selection favoring carnivores may be widespread (Figure 2; Table 1). Yet, if one extreme morph (the carnivore morph) has higher fitness than the other extreme morph (the omnivore morph), how is resource polymorphism maintained in this system?

Several factors examined in previous studies may help maintain both omnivores and carnivores within the same population. First, as carnivores become increasingly common in any given pond, and as competition among carnivores for the limited shrimp resource thereby becomes more intense, negative frequency-dependent selection favors omnivores [43]. In support of these earlier findings, we found evidence in this study for pure disruptive selection (with no directional component) in two ponds, and disruptive selection with a directional component favoring *omnivores* in another (Figure 2; Table 1). Moreover, although carnivores benefit by gaining access to the more profitable shrimp resource (which could explain why they often achieve larger body size), additional experiments have shown that carnivores also experience greater competition with other carnivores than omnivores do with other omnivores [37]. In this way, negative frequency-dependent selection maintains *both* morphs within the same population [43]. Second, fitness trade-offs are associated with each of these phenotypic alternatives. Compared to carnivores, omnivores invest more into abdominal fat bodies, which increases post-metamorphic resistance to starvation [43]. Thus, although carnivores may often have higher survival before metamorphosis (the life stage at which we estimated selection in the present study), omnivores may have higher survival immediately *after* metamorphosis, possibly balancing their lower pre-metamorphic survival [43]. Generally, frequency-dependent selection, coupled with fitness trade-offs, likely contribute to the evolutionary maintenance of many resource polymorphisms [4].

A deeper problem requiring explanation, however, is why intermediate phenotypes persist in the face of widespread disruptive selection against them (Figure 1). Although morph determination is environmentally triggered—carnivore development is induced when a tadpole consumes fairy shrimp [50]—considerable heritable variation in the response to this cue exists in natural populations [48,51], suggesting that the propensity to produce intermediate phenotypes could, in principle, be eliminated by natural selection. A possible resolution to this problem is that sexual selection may lead to random mating in regard to larval phenotype, recreating tadpoles likely to develop as intermediate phenotypes in each generation. Specifically, in our populations, female *S. multiplicata* prefer to mate with males in good condition, irrespective of the females’ own condition [54,55]. Therefore, female *S. multiplicata* will tend to mate with males of good condition, potentially regardless of either the male’s larval phenotype or the female’s own larval phenotype (e.g., a good-condition adult male might have been either an omnivore or a carnivore as a tadpole). This directional selection on male condition (exerted by female preferences for mates) might thereby oppose disruptive selection acting on the larvae. Indeed, directional sexual selection may often oppose disruptive natural selection in many populations [56], which may explain why an increasing number of cases have been reported in which assortative mating by resource-use phenotype was expected but not found [57,58]*.*

Spatial and temporal variation in the mode of selection may also help explain the persistence of intermediate larval phenotypes. For example, stabilizing selection favoring individuals with intermediate phenotypes was detected in some ponds (Figure 1, Figure 2), specifically those lacking strong intraspecific competition (Figure 3A3B). Stabilizing selection was also detected in ponds in which *S. multiplicata* co-occurs with a congener, *Spea bombifrons *[21]. Such mixed-species ponds may be within a few kilometers of pure *S. multiplicata* ponds in the San Simon Valley of southeastern Arizona [53]. Thus, *S. bombifrons* migrants may occasionally colonize nearby pure *S. multiplicata* ponds, thereby changing the selective regime within the pond to one in which intermediate phenotypes are favored. Indeed, *S. bombifrons* occurred along with *S. multiplicata* within some of the ponds sampled for this study as recently as 30 years ago [59]. Thus, because the mode and direction of selection is spatially and temporally variable, intermediate phenotypes may persist in the population.

Finally, limits in the ability of tadpoles to respond appropriately through phenotypic plasticity to variation in both fairy shrimp density [50] and their competitive environment (i.e., conspecific density and morph frequency) may explain the persistence of intermediate phenotypes in natural ponds. For instance, spatial and temporal variation in the density of fairy shrimp within a pond could give some individuals a head start in carnivore expression early in development. Once tadpoles increase their mobility as they grow and develop, tadpoles encountering fairy shrimp later may begin to develop as carnivores, but these late-developing carnivores might end up being outcompeted by earlier-developing carnivores. If these individuals are deprived of fairy shrimp, their carnivore-like features begin to regress, and they assume an intermediate phenotype [43]. Generally, intermediate phenotypes may be difficult to eradicate via disruptive selection when limits exist in the ability of individuals to assess and respond adaptively to their competitive environment though phenotypic plasticity*.* Further studies are needed to test these ideas.

## Conclusions

Although disruptive selection has long been viewed as a potential generator of biological diversity, it has received relatively little attention, and, consequently, little is known regarding its prevalence or causes within specific systems. Using spadefoot toad tadpoles as a model system, we found that disruptive selection can be prevalent in the wild, and that its occurrence is predictably associated with a specific ecological factor: strong intraspecific competition. Given that intraspecific competition is common in many natural populations—and frequently strong [60,61]—our results therefore imply that disruptive selection may be a more important force contributing to the origin and maintenance of biological diversity than is currently appreciated.

## Methods

All procedures were carried out in compliance with the Institutional Animal Care and Use Committee at the University of North Carolina under application 06-047.0-A. Field collections were conducted under New Mexico collecting permit 1857 and Arizona collecting permit SP604848.

### Field surveys

We collected *S. multiplicata* tadpoles during summers 2006-2009 from 15 ephemeral ponds in the San Simon Valley (Figure 1). In all 15 ponds, *S. multiplicata* was the only species of *Spea* present. Six ponds were sampled across multiple years for a total of 22 collections (Figure 1; Table 2). We sampled each pond on a single day 16-20 days after breeding had occurred (*Spea* breed only a single time within a pond each season, shortly after the pond fills with rainwater). Within each pond, we sampled tadpoles from five randomly selected sites throughout the pond using a hand-held dip net (median sample size = 130 tadpoles). We sacrificed the tadpoles immediately after collection by immersion in a 0.1% aqueous solution of tricane methanesulfonate (MS 222) and preserved them in 95% ethanol. We used this random sampling technique to estimate the density of *S. multiplicata* tadpoles in each pond as ‘high’, ‘moderate’ and ‘low’ [46]. Additionally, we determined the range of available resources in each pond by estimating abundances of fairy shrimp and detritus, which are the two main resources on which *Spea* tadpoles feed. We estimated fairy shrimp abundance by sweeping a net throughout each pond and categorizing shrimp densities as “high,” “moderate,” and, “low”. These subjective estimates were corroborated by previously published intensive, quantitative sampling [50,53]. We assessed the availability of detritus by estimating the percent vegetative cover in a twenty meter radius around each pond’s circumference and categorized each pond as having either “high” (67%-100% cover), “moderate” (34%-66% cover) or, “low” (0%-33% cover) detritus (ponds with more vegetation tend to have more detritus; see [53]).

**Table 2:** Summary of tadpole collections and ecological parameters in natural ponds

**Pond**	**Map ID**	**Year**	**N**	**Tadpole density**	**Fairy shrimp density**	**Cover (%)**
AZ0602	1	2006	93	2	3	.8
AZ0603	2	2006	124	1	2	.75
AZ0604	3	2006	50	2	3	.5
AZ0605	4	2006	176	3	2	.6
AZ0606	5	2006	94	2	1	.9
AZ0607	6	2006	102	2	1	.4
NM0608	7	2006	165	3	2	1
AZ0710	8	2007	78	3	1	.9
AZ0711	9	2007	99	2	3	1
AZ0706	10	2007	125	1	2	.7
AZ0801	11	2008	99	1	2	1
AZ0810	12	2008	213	3	2	.8
AZ0811	13	2008	181	3	3	.5
AZ0816	14	2008	297	3	2	1
AZ0809	15	2008	59	3	3	1
AZ0802	16	2008	150	2	2	.7
AZ0812	17	2008	188	2	2	.6
AZ0813	18	2008	135	2	2	1
NM0810	19	2008	169	2	2	.8
AZ0903	20	2009	78	3	1	.75
AZ0902	21	2009	211	3	3	1
AZ0904	22	2009	192	1	1	1

For each population the pond name, map ID corresponding to Figure 1, sampling year, tadpole sample size (N), tadpole density, fairy shrimp density, and percentage of vegetative cover (Cover) are shown. We assigned numerical values to our estimates of tadpole and shrimp abundance such that “high” = 3, “medium” = 2 and “low” = 1.

We calculated an estimate of per capita resource abundance for each pond by dividing the sum of fairy shrimp and detritus abundance (“high” = 3, “medium” = 2 and “low” = 1) by *S. multiplicata* tadpole density (“high” = 3, “medium” = 2 and “low” = 1). We subsequently used this score to test our prediction that ponds with high levels of intraspecific competition experienced the most intense disruptive selection.

### Evaluating the prevalence of disruptive selection

To evaluate the prevalence of disruptive selection on trophic morphology across ponds, we first calculated a composite index of trophic morphology, separately for each pond, following previously described methods [18,21,46]. Briefly, we began by measuring each tadpole’s snout-vent-length (SVL) using hand-held digital calipers. For each tadpole, we additionally measured the width of the orbitohyoideus (OH) muscle and characterized the shape of each tadpole’s keratinized mouthparts (MP) on an ordinal scale from one (most omnivore-like) to five (most carnivore-like), and counted the number of denticle rows (DR) surrounding the keratinized mouthparts. We standardized OH for body size (SVL) by regressing ln (i.e., natural log) OH on ln SVL and used the resulting residuals for the subsequent analyses (these residuals were distributed normally). We then combined the MP scores, DR counts, and residuals of ln OH regressed on ln SVL, into a single multivariate shape variable (the “morphological index”; see [18]) with a principal component analysis using the correlation matrix, and standardized to have a standard deviation of one. Our morphological index for each pond was the first principal component. Larger values of PC1 correspond to more carnivore-like tadpoles, with larger OH muscles (corrected for body size), fewer denticle rows, and more serrated, notched mouthparts. In contrast smaller values of PC1 correspond to more omnivore-like tadpoles, with smaller OH muscles (corrected for body size), more denticle rows and smooth mouthparts (Table 3).

**Table 3:** Principal component analysis of trophic morphology

**Pond**	**Map ID**	**PC**	**MP eigenvector loading**	**residual OH eigenvector loading**	**DR eigenvector loading**	**% variance explained**
AZ0602	1	1	.675	.692	-.252	49.5
		2	-.252	-.104	-.961	32.4
		3	-.692	.713	.104	18.0
AZ0603	2	1	.707	.707	—	82.0
		2	.707	-.707	—	17.9
		3	—	—	—	—
AZ0604	3	1	.594	.647	-.477	62.5
		2	-.511	-.154	-.845	25.7
		3	.620	-.746	-.238	11.6
AZ0605	4	1	.576	.644	-.502	59.9
		2	-.581	-.108	-.806	25.9
		3	.574	-.756	-.312	14.2
AZ0606	5	1	.707	.707	—	76.5
		2	.707	-.707	—	23.5
		3	—	—	—	—
AZ0607	6	1	.691	-.466	-.372	52.2
		2	-.057	-.057	-.882	31.7
		3	-.720	-.882	-.287	15.7
NM0608	7	1	.701	.690	-.176	52.3
		2	.058	-.190	-.979	32.8
		3	.710	.697	-.093	14.7
AZ0710	8	1	.600	.591	-.538	64.0
		2	-.318	-.441	-.838	20.8
		3	-.733	.675	-.076	15.14
AZ0711	9	1	.590	.475	-.652	64.3
		2	.530	-.837	-.130	25.7
		3	-.608	-.269	-.746	9.8
AZ0706	10	1	.560	.608	-.561	71.0
		2	.712	-.007	.701	17.8
		3	-.423	.793	.438	11.1
AZ0801	11	1	.534	.589	-.605	44.8
		2	.836	-.470	.279	28.7
		3	-.120	-.656	-.745	26.3
AZ0810	12	1	.588	.547	-.594	68.0
		2	.442	-.833	-.330	18.4
		3	.676	.068	.732	13.4
AZ0811	13	1	.597	.590	-.542	53.5
		2	-.327	-.437	-.837	24.9
		3	.731	-.678	.068	21.5
AZ0816	14	1	.597	.551	-.581	66.3
		2	.242	-.815	-.525	19.0
		3	-.764	.172	-.621	14.5
AZ0809	15	1	.593	.558	-.578	49.4
		2	-.211	.802	.557	26.1
		3	-.776	.208	-.595	24.4
AZ0802	16	1	.628	.684	-.369	55.9
		2	-.429	-.090	-.898	30.9
		3	-.648	.723	.237	13.1
AZ0812	17	1	.641	.501	-.580	59.9
		2	-.125	.815	.565	25.6
		3	.756	-.290	.585	14.4
AZ0813	18	1	.550	.777	-.304	38.7
		2	-.607	.122	-.784	36.6
		3	-.572	.617	.539	24.6
NM0810	19	1	.577	.595	-.558	76.0
		2	-.571	-.192	-.797	14.3
		3	.582	-.779	-.229	9.6
AZ0903	20	1	.404	.710	-.576	47.5
		2	.814	.006	.579	33.5
		3	-.415	.703	.576	18.6
AZ0902	21	1	.591	.556	-.583	76.5
		2	.307	-.824	-.475	14.0
		3	.745	-.101	.658	9.4
AZ0904	22	1	.557	.588	-.585	49.2
		2	.828	-.350	.436	26.1
		3	-.051	.728	.683	24.5

For each population the pond name, map ID corresponding to Figure 1, principal component axis (PC), trait loadings and % variance explained are shown.

We were unable to obtain denticle row counts for tadpoles from two collections (AZ0603 and AZ0606, see Table 3). Therefore, for these two collections we calculated an alternative morphological index using only MP scores and residual values of ln OH corrected for ln SVL [21]. We calculated a single multivariate shape variable by combining the MP scores and residuals OH values by again using a principal component analysis as described above. As before, the morphological index for each of the two ponds was the first principal component. Larger values of PC1 correspond to more carnivore-like tadpoles, with larger OH muscles (corrected for body size), and more serrated, notched mouthparts. In contrast smaller values of PC1 correspond to more omnivore-like tadpoles, with smaller OH muscles (corrected for body size), and smooth mouthparts (Table 3).

We estimated the mode and magnitude of selection acting on tadpole trophic morphology in each pond using body size as a fitness proxy [ln SVL; see 21, 62]. Body size is positively correlated with fitness in many species [62], including in *Spea*. For example, relative to tadpoles within the same population that are larger*,* tadpoles that are smaller in body size have a lower probability of survival, both before [63] and after metamorphosis [64]. Moreover, smaller tadpoles tend to be less developmentally advanced, and there is a premium on rapid development in the ephemeral ponds in which *Spea multiplicata* typically breed [43]. Additionally, in *Spea,* adult size is positively correlated with mating success in males [54] and fecundity in females [63]. Finally, a previous field experiment, in which tadpoles of different morphotypes were marked and recaptured within a natural pond, established that the relationship between morphology and body size mirrored that between body size and *survival *[18]. In short, body size is a reliable proxy for fitness in this system.

To estimate the magnitude and mode of selection, we ran separate linear and quadratic regression (including both linear and quadratic terms for the latter) of relative fitness (ln SVL/mean ln SVL) onto the morphological index for each pond [65,66]. We obtained standardized selection differentials for linear and quadratic selection from these regressions of relative fitness on trophic morphology. We doubled the quadratic regression coefficients to obtain quadratic selection differentials (γ) [66,67].

A significant, standardized linear selection differential (ß) indicates that directional selection is acting on trophic morphology [66]. Selection on trophic morphology might be disruptive when the quadratic selection differential (γ) is significantly positive, and stabilizing when it is negative [66]. However, a significant γ is necessary, but not sufficient to indicate the presence of quadratic selection where intermediate phenotypes are at the fitness maximum/minimum [68]. Therefore, for each pond, we fit cubic splines between the morphological index and fitness along with 95% confidence intervals to visualize the selective surface [69]. Cubic spline analysis is less sensitive to outliers and allows estimation of a fitness function without an *a priori* assumption about the function’s shape [69]. We visually inspected these plots to determine if there was an intermediate fitness minimum or maximum within the range of the data (Figure 2). For the ponds with significant quadratic regression differentials we also applied a constrained regression method to statistically evaluate if the null hypotheses that fitness minimum/maximum lie at extreme phenotypic values (rather than within the observed range of the data) can be rejected (Table 4) [68,70]. Where the graphical and statistical methods conflicted (in two cases) we favored our visual evaluation of the cubic splines because the constrained regression method is sensitive to deviations from the assumptions of parametric statistical tests that do not affect the nonparametric cubic spline approach [69].

**Table 4:** Mitchell-Olds and Shaw constrained regression tests for fitness minimum/maximum indicating quadratic selection

**Pond**	**Map ID**	***F***_***min***_	***P***_***min***_	***F***_***max***_	***P***_***max***_	**fitness min/max**
AZ0604	3	5.124	.028	10.522	.002	Y
AZ0605	4	48.257	<.0001	80.282	<.0001	Y
AZ0607	6	12.177	.0007	5.311	.024	Y
NM0608	7	7.810	.005	16.573	<.0001	Y
AZ0710	8	12.492	.0006	30.103	<.0001	Y
AZ0711	9	28.468	<.0001	1.559	.214	N
AZ0801	11	31.686	<.0001	8.232	.005	Y
AZ0810	12	23.971	<.0001	61.126	<.0001	Y
AZ0811	13	21.384	<.0001	38.224	<.0001	Y
AZ0816	14	85.242	<.0001	172.109	<.0001	Y
AZ0802	16	24.155	<.0001	10.773	.001	Y
AZ0812	17	20.838	<.0001	36.537	<.0001	Y
AZ0813	18	5.154	.024	14.562	.0002	Y
NM0810	19	10.912	.001	16.500	<.0001	Y
AZ0903	20	.083	.773	.317	.574	N
AZ0902	21	1.691	.194	14.560	.0001	N
AZ0904	22	39.010	<.0001	9.252	.002	Y

For each population the pond name, map ID corresponding to Figure 1, test statistics and results evaluating if the null hypotheses that fitness minimum/maximum lie at extreme minimum and maximum phenotypic values (rather than within the observed range of the data) can be rejected.

### Evaluating whether competition predicts the strength of disruptive selection

Finally, we sought to test our prediction regarding the ecological correlates of disruptive selection. Specifically, we predicted that disruptive selection would be strongest in ponds with intense intraspecific competition. To test this prediction, we fit separate linear regressions of the quadratic selection differentials onto two estimates of intraspecific competition: (1) conspecific density, and (2) per capita resource density. Our underlying assumption was that intraspecific competition would be more intense the higher the density of conspecifics and the lower the per capita resource density within a pond.

We weighted the selection differentials in each regression described above by the square root of our sample size for each pond. We did so because confidence in the estimation of both the sign and magnitude of selection differentials is lower for those ponds with small sample sizes then for those with larger sample sizes.

To further evaluate our models we present confidence intervals obtained from bootstrapping for each analysis to account for potential pseudo-replication introduced by using temporal replicates from the same pond. For the regression models used in testing our two predictions, we sampled a single selection differential from each of the fifteen unique ponds, fit the regression, and extracted the regression coefficients. We resampled the selection differentials and refit each model 1000 times and then estimated 95% confidence intervals for each regression coefficient. We assessed significance by asking if the range of the confidence interval excluded zero. All statistical analyses were performed using R (version 2.15.0) [71].

## Competing interests

The authors declare that they have no competing interests.

## Authors’ contributions

RM and DP designed the study, did field work, and wrote the paper. RM analyzed the data. Both authors approved the final version of the manuscript.
